# Impact of left atrial anatomy on pulmonary vein isolation with cryoballoon ablation: Insights from the randomized controlled COMPARE CRYO study

**DOI:** 10.1016/j.hroo.2025.07.005

**Published:** 2025-07-16

**Authors:** David Spreen, Thomas Kueffer, Salik ur Rehman Iqbal, Patrick Badertscher, Jens Maurhofer, Philipp Krisai, Corinne Isenegger, Behnam Subin, Nicolas Schärli, Beat Schaer, Vincent Schlageter, Maurice Pradella, Corinne Jufer, Gregor Thalmann, Helge Servatius, Hildegard Tanner, Felix Mahfoud, Michael Kühne, Laurent Roten, Tobias Reichlin, Christian Sticherling, Sven Knecht

**Affiliations:** 1 University Heart Center, University Hospital Basel, University Basel, Basel, Switzerland; 2 CRIB Cardiovascular Research Institute Basel, University Hospital Basel, University of Basel, Basel, Switzerland; 3 Department of Cardiology, Inselspital - University Hospital Bern, Bern, Switzerland; 4 Department of Radiology, University Hospital Basel, University Basel, Basel, Switzerland

**Keywords:** Left atrium, Pulmonary vein isolation, Cryoballoon ablation, Atrial fibrillation, Anatomical features, Anatomy, Recurrence, First-pass isolation

## Abstract

**Background:**

Cryoballoon ablation is an established therapy for pulmonary vein (PV) isolation (PVI).

**Objective:**

This study aimed to explore whether specific left atrial anatomical features are associated with both first-pass PVI success and long-term outcomes after cryoballoon ablation using 2 different cryoablation systems.

**Methods:**

Left atrial reconstructions of patients with paroxysmal atrial fibrillation were analyzed. PVI was performed using either the Medtronic (Minneapolis, MN) Arctic Front Advance or the Boston Scientific (Marlborough, MA) POLARx cryoablation catheter. Anatomical features were assessed to predict first-pass PVI success after a single application and long-term outcomes on the basis of implantable cardiac monitor recordings.

**Results:**

A total of 191 patients were enrolled (mean age 63.0 ± 9.8 years; 58 (30%) women). First-pass PVI was achieved in 69% of all procedures. Female sex was significantly associated with reduced first-pass isolation success, limited to the right superior PV (odds ratio [OR] 0.50; 95% confidence interval [CI] 0.26–0.97; *P* = .04). Anatomical predictors of failure of first-pass PVI included the absence of an orthogonal orientation of the left superior PV (OR 0.20; 95% CI 0.05–0.88; *P* = .033) and the left inferior PV (OR 0.36; 95% CI 0.13–0.99; *P* = .047) as well as the presence of a right middle PV for the right superior PV (OR 3.58; 95% CI 1.18–10.9; *P* = .024). The absence of an orthogonal orientation of the left superior PV was associated with atrial tachyarrhythmia recurrence (OR 4.12; 95% CI 1.90–9.11; *P* < .001).

**Conclusion:**

The absence of an orthogonal orientation of the left-sided PVs was significantly associated with lower first-pass isolation rates and a higher risk of recurrence. These findings highlight the importance of preprocedural anatomical assessment to identify potential challenges and tailor ablation strategies.

## Graphical abstract

**Figure:**
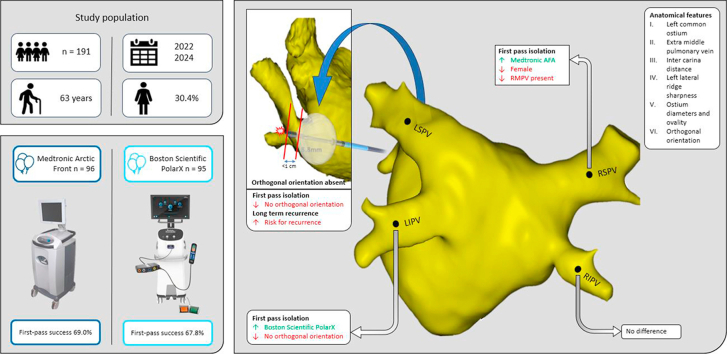

**Key Findings**

▪ Anatomical parameters can predict both acute and long-term pulmonary vein isolation (PVI) success.
▪ The absence of an orthogonal orientation of the left superior pulmonary vein (LSPV) and left inferior pulmonary vein was identified as a significant predictor of reduced first-pass PVI success.
▪ The absence of an orthogonal orientation of the LSPV increases the risk of arrhythmia recurrence after 1 year.
▪ Female sex was associated with lower first-pass isolation rates in the right superior pulmonary vein.

## Introduction

For the treatment of atrial fibrillation (AF), pulmonary vein (PV) isolation (PVI) remains the cornerstone of catheter ablation.^1^^,^^2^ Previous studies have shown that cryoballoon (CB) ablation (CBA) is noninferior to radiofrequency ablation.^3^, ^4^, ^5^ In contrast to the common steerable focal radiofrequency catheter, which has a small 7.5-F tip diameter and allows the creation of flexible lesion sets within the left atrium (LA), the CB with a circular diameter of 28 or 31 mm is specifically designed to perform circumferential PVI. Because of the anatomical variability of the LA and PVs, previous studies have investigated the impact of anatomical features on both the short- and long-term success of the procedure.^6^, ^7^, ^8^, ^9^ In these studies, parameters such as LA volume, PV ostium diameter, ridge ratios, branching features, PV circumferences, and ovality have been evaluated for their influence on procedural outcomes using the Medtronic CB catheter (Arctic Front Advance, Medtronic, Minneapolis, MN). Even with the availability of a second CBA system (POLARx, Boston Scientific, Marlborough, MA), it remains unclear whether these established predictors still apply or whether new anatomical factors may influence its efficacy. This study was designed as an exploratory analysis to identify potential associations between LA anatomy and both first-pass PVI success and arrhythmia recurrence after CBA.

## Methods

### Study population

We retrospectively analyzed data from the randomized controlled COMPARE CRYO (Comparison of PolarX and the arctic front cryoballoons for PVI in patients with symptomatic paroxysmal AF) study, which investigated the efficacy and safety of the POLARx CB catheter (POLARx group) compared with the Arctic Front Advance CB catheter (AFA group) for PVI in patients with paroxysmal AF.^4^ Patients with persistent AF, previous LA ablation or surgery, AF owing to reversible causes, left ventricular ejection fraction < 35%, or New York Heart Association functional class III/IV heart failure were excluded. All patients were implanted with an implanted cardiac monitor at the end of the procedure.

### PVI

PVI was performed as described in detail elsewhere.^4^ Briefly, the CB was introduced into the LA after fluoroscopy-guided transseptal puncture and placed at the ostium of the PVs. PV occlusion was determined using contrast injection, and *acute ablation success* was defined as the disappearance of local PV signals on the circular mapping catheters from within the PVs or temperature drops below −50°C and −40°C within 60 seconds for the POLARx and AFA groups, respectively. In case of an ineffective freeze, the balloon was repositioned, and a new lesion was created. Phrenic nerve pacing and monitoring were performed during ablation of the right-sided PVs to prevent phrenic nerve palsy (PNP).^10^

### Preprocedural imaging

Cardiac magnetic resonance imaging (MRI) was performed on a 1.5-T scanner (Magnetom Avanto/Espree, Siemens Healthineers, Erlangen, Germany). A T1-weighted fast low-angle shot spoiled gradient echo sequence (repetition time 3.7 seconds; echo time 1.3 ms; flip angle 25°; bandwidth 63.6 kHz), with images reconstructed to a 512 × 512 matrix and a voxel size of 2.25 × 2.25 × 1.2 mm), was acquired in a coronal plane before and after contrast injection (0.1 mmol/kg at 2 mL/s, MultiHance, Bracco, Milano, Italy).

Preprocedural cardiac computed tomography (CT) scans were electrocardiogram-triggered on the Siemens SOMATOM Definition Flash CT scanner during inspiratory breath-hold. Contrast injection (90 mL of Ultravist-370, Bayer Healthcare, Berlin, Germany) was administered via the left brachial vein at 4.5 mL/s, followed by a 20-mL saline chaser; this was repeated after 3 minutes. Bolus tracking was triggered on the ascending aorta, and images were reconstructed into 0.75-mm axial sections.

### Anatomical parameters

**Figure 1:**
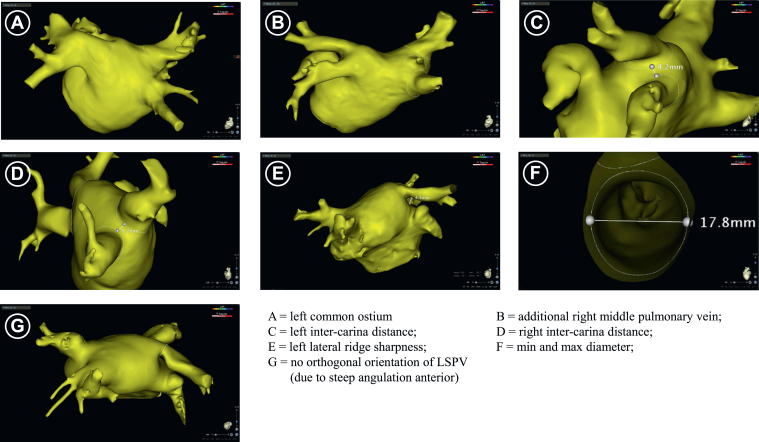
Visualization matrix of the investigated anatomical features. LSPV = left superior pulmonary vein; max = maximum; min = minimum.

All anatomical structures were segmented and reconstructed in CARTO 3 (CartoSeg, Biosense Webster, Diamond Bar, CA). The endocardial surface of the LA was visualized, and the PV ostia were manually delineated at the visual intersection of the PVs with the LA (Figure 1A–1G), based on the inflection between the LA and PV walls.^7^^,^^11^ A left common ostium (LCO) was defined on the basis of the length of at least 10 mm of the anterior ridge from the LA ostium to the PV bifurcation (Figure 1A). The presence of a right middle PV (RMPV) was assessed visually (Figure 1B). An *RMPV* was strictly defined as a distinct additional vein with a clearly separated ostium connecting directly to the LA.^11^ The *left and right carina distances*, defined as the shortest distances between both carinae of the ipsilateral PVs, were measured using the electronic calliper (Figure 1C and 1D). For CT images only, the sharpness of the left lateral ridge between the left superior and inferior PVs (LSPV/LIPV) and the LA appendage was determined (Figure 1E) on the basis of the local curvature <0.5 1/mm, reflecting a local radius of >2 mm.^6^ To assess the maximum and minimum diameters of the PVs, a clipping plane parallel to the plane of each ostium was placed. The maximum and minimum diameters were used to calculate PV ovality by dividing the minimum diameter by the maximum diameter (Figure 1F). PV orientation was assessed on the basis of the presence of an orthogonal isocentric line on the ostial clipping plane, with a length of at least 1 cm before intersecting the endoluminal PV surface (Figure 1G and Supplemental Figure 1). This anatomical configuration allows an unimpaired orientation of the balloon catheter in the PV.

### End points

The primary end point of first-pass isolation was defined as acute PVI of each individual PV with the first application according to the above-defined criterium. The secondary end point was the first recurrence of any atrial tachyarrhythmia (AF, atrial flutter, or atrial tachycardia) between days 91 and 365 postablation, detected by an implantable cardiac monitor. A subanalysis comparing differences between the 2 systems in first-pass PVI was performed (Supplemental Table 1).

**Table 1:** Baseline characteristics

Characteristic	All (N = 191)	AFA group (n = 96)	POLARx group (n = 95)
Age (y)	63.0 ± 9.8	62.9 ± 9.4	63.1 ± 10.2
Female sex	58 (30.4)	23 (24.0)	35 (36.8)
BMI (kg/m^2^)	26.8 ± 4.5	26.6 ± 4.2	26.9 ± 4.9
Medical			
Hypertension	98 (51.3)	52 (54.2)	46 (48.4)
Diabetes mellitus	13 (6.8)	6 (6.3)	7 (7.4)
History of stroke	12 (6.3)	4 (4.2)	8 (8.4)
CHA_2_DS_2_-VASc score	1.72 ± 1.5	1.6 ± 1.4	1.8 ± 1.6
OSAS	14 (7.3)	6 (6.3)	8 (8.4)
Echocardiography			
LA diameter (mm)	39.9 ± 6.4	40.3 ± 6.3	39.6 ± 6.6
LVEF (%)	60.6 ± 5.5	61.0 ± 5.5	60.4 ± 5.1
LAVI (mL/m^2^)	34.5 ± 11.0	34.4 ± 9.8	34.3 ± 11.7

Continuous variables are presented as mean ± standard deviation and categorical parameters as n (%).

AFA = arctic front advance; BMI = body mass index; LA = left atrial; LAVI = left atrial volume index; LVEF = left ventricular ejection fraction; OSAS = obstructive sleep apnea syndrome.

### Data analysis

Continuous data are described as mean *±* standard deviation or median with interquartile range, as appropriate. Categorical data are described as absolute number and proportion. Comparisons of the characteristics were performed using an unpaired Student *t* test or the Mann-Whitney *U* test for continuous variables, depending on normality, and the χ^2^ test or Fisher exact test for categorical variables.

A univariate logistic regression analysis was performed per-vein and per-ablation system. For the secondary end point, a Cox regression analysis with stepwise forward selection was performed to identify anatomical predictors of recurrence. For the LCO without separate left-sided PV features, the analysis was performed twice: once excluding patients with LCO but including all anatomical features, and once including all patients but excluding data related to the LIPV, assuming that LSPV parameters are equal to left common PV parameters.

The intra-observer reliability of the measurements was assessed on the basis of repeated measurements of a random subset of 10 patients. The absolute and relative differences between both measurements were calculated for each feature, and the mean and standard deviation of these differences are reported to quantify measurement variability.

All statistical analyses were performed using SPSS version 23.0 (IBM Corporation, Armonk, NY), and a *P* value of <.05 was considered statistically significant.

## Results

### Baseline characteristics

Of the included 202 patients, 102 were randomized to the AFA group and 100 to the POLARx group. After excluding 11 patients because of low image quality (in 10 patients) or consent withdrawal (in 1 patient), images from 191 patients were analyzed (Table 1). Of these patients, 129 had CT scans (61%) and 62 had MRI scans (39%). PVI was achieved in all veins with 1.4 ± 0.5 applications, with no difference between the groups. Baseline and procedural characteristics are presented in Table 1, Table 2 and Table 1, Table 2.

**Table 2:** Procedural characteristics

Characteristic	All (N = 191)	AFA group (n = 96)	POLARx group (n = 95)	*P*
General anesthesia	8 (4.2)	5 (5)	3 (3)	.72
Duration (min)	70.5 ± 26.2	68.5 ± 25.3	72.5 ± 27.1	.28
LA dwell (min)	48.0 ± 17.4	46.8 ± 16.4	49.2 ± 18.4	.34
Fluoroscopy				
Time (min)	15.6 ± 7.7	15.9 ± 8.0	15.3 ± 7.5	.62
Dose (μGy·m^2^)	678 ± 781	740 ± 878	616 ± 669	.28
Applications				
LSPV	1 (1–2)	1 (1–2)	1 (1–1)	na
LIPV	1 (1–1)	1 (1–2)	1 (1–1)	na
RSPV	1 (1–2)	1 (1–1)	1 (1–2)	na
RIPV	1 (1–2)	1 (1–2)	1 (1–2)	na
Number	5 (4–7)	5 (4–7)	5 (4–7)	na
Time (s)	1015 ± 329	1027 ± 322	1002 ± 336	.61
First-pass vein isolation				
LSPV (n = 191)	140 (73)	68 (71)	72 (76)	.44
LIPV (n = 180)	133 (70)	60 (63)	73 (77)	.03
RSPV (n = 188)	135 (71)	76 (79)	59 (62)	.01
RIPV (n = 191)	127 (67)	60 (63)	67 (71)	.24
Total		264 (69)	271 (72)	

Values are presented as mean ± standard deviation, median (interquartile range), or n (%).

AFA = arctic front advance; LA = left atrial; LIPV = left inferior pulmonary vein; LSPV = left superior pulmonary vein; na = not applicable; RIPV = right inferior pulmonary vein; RSPV right superior pulmonary vein.

### End points

#### Impact of atrial anatomy on first-pass PVI success

Except for female sex affecting isolation of the right superior PV (RSPV) (odds ratio [OR] 0.50; 95% confidence interval [CI] 0.26–0.97; *P* = .04), none of the baseline characteristics were identified as predictors of first-pass PVI (Table 3). The maximum diameter of the RSPV was significantly smaller in women than in men (22.8 ± 2.97 mm vs 23.6 ± 3.41 mm; *P* = .011). The absence of an orthogonal orientation of the LSPV and LIPV was identified as a predictor of first-pass PVI failure (OR 0.20; 95% CI 0.05–0.84; *P* = .03 and OR 0.36; 95% CI 0.13–0.99; *P* = .05, respectively). The presence of an RMPV was significantly associated with lower first-pass isolation rate for the RSPV (OR 3.6; 95% CI 1.2–10.9; *P* = .02) (Table 4).

**Table 3:** Logistic regression per vein for baseline parameters associated with first-pass success

Variable	OR	CI	Significance
Age			
Whole cohort	0.992	0.961–1.024	.622
LSPV	0.999	0.966–1.032	.943
LIPV	0.989	0.958–1.021	.494
RSPV	0.994	0.963–1.027	.728
RIPV	0.979	0.948–1.010	.181
Female sex			
Whole cohort	0.863	0.435–1.713	.674
LSPV	0.938	0.496–1.875	.855
LIPV	1.370	0.686–2.733	.372
RSPV	0.502	0.260–0.969	**.040**
RIPV	0.678	0.357–1.289	.236
BMI			
Whole cohort	0.990	0.923–1.062	.786
LSPV	1.023	0.952–1.100	.532
LIPV	0.950	0.888–1.016	.133
RSPV	0.980	0.916–1.049	.564
RIPV	0.985	0.922–1.052	.654
LA diameter			
Whole cohort	0.956	0.907–1.007	.089
LSPV	1.003	0.952–1.058	.899
LIPV	0.953	0.906–1.004	.068
RSPV	0.969	0.920–1.022	.247
RIPV	0.966	0.920–1.016	.178
LVEF			
Whole cohort	0.993	0.936–1.053	.808
LSPV	0.968	0.911–1.029	.291
LIPV	1.026	0.971–1.086	.361
RSPV	0.977	0.921–1.035	.426
RIPV	0.992	0.939–1.049	.788
LAVI			
Whole cohort	0.980	0.951–1.011	.207
LSPV	1.006	0.975–1.038	.716
LIPV	0.982	0.955–1.011	.220
RSPV	0.997	0.967–1.027	.820
RIPV	0.996	0.968–1.025	.793

Significant *P* values are highlighted in bold.

BMI = body mass index; CI = confidence interval; LA = left atrial; LAVI = left atrial volume index; LIPV = left inferior pulmonary vein; LSPV = left superior pulmonary vein; LVEF = left ventricular ejection fraction; OR = odds ratio; RIPV = right inferior pulmonary vein; RSPV right superior pulmonary vein.

**Table 4:** Logistic regression per anatomical feature for all veins associated with first-pass success

Variable	OR	CI	Significance
LSPV			
Left common ostium	1.205	0.458–2.294	.952
Inter-carina distance	1.164	0.927–1.461	.191
Lateral ridge sharpness	1.070	0.384–2.978	.897
Min diameter	1.013	0.944–1.088	.710
Max diameter	0.995	0.928–1.066	.879
Ovality	1.010	0.984–1.036	.462
No orthogonal orientation	0.201	0.046–0.876	**.033**
LIPV			
Inter-carina distance	1.012	0.838–1.221	.903
Lateral ridge sharpness	1.071	0.399–2.875	.891
Min diameter	1.033	0.928–1.151	.548
Max diameter	1.016	0.907–1.139	.783
Ovality	1.008	0.979–1.038	.613
No orthogonal orientation	0.364	0.134–0.988	**.047**
RSPV			
Right middle pulmonary vein	3.583	1.182–10.86	**.024**
Inter-carina distance	1.085	0.957–1.231	.200
Min diameter	1.080	0.984–1.186	.104
Max diameter	1.049	0.954–1.153	.324
Ovality	1.019	0.991–1.049	.190
No orthogonal orientation (n = 0)	na	na	na
RIPV			
Right middle pulmonary vein	2.881	0.954–8.697	.061
Inter-carina distance	1.014	0.903–1.138	.817
Min diameter	0.943	0.859–1.034	.210
Max diameter	0.981	0.901–1.067	.650
Ovality	0.981	0.947–1.018	.311
No orthogonal orientation (n = 0)	na	na	na

Significant *P* values are highlighted in bold.

CI = confidence interval; LIPV = left inferior pulmonary vein; LSPV = left superior pulmonary vein; Max = maximum; Min = minimum; na = not applicable; OR = odds ratio; RIPV = right inferior pulmonary vein; RSPV right superior pulmonary vein.

In a per-system analysis, first-pass PVI was achieved in 69% and 72% of all veins in the AFA and POLARx groups, respectively (Table 2). In a per-vein analysis, first-pass PVI in the POLARx group was significantly higher in the LIPV (*P* = .03) and significantly lower in the RSPV (*P* = .01) compared with the AFA group (Table 2). The absence of an orthogonal orientation of the LSPV was identified as a significant predictor only when using the POLARx catheter (OR 0.08; 95% CI 0.008–0.833; *P* = .035) (Supplemental Table 1).

#### Impact of atrial anatomy on long-term recurrence after PVI

The absence of an orthogonal orientation of the LSPV was associated with an increased risk of recurrence within 1 year (OR 2.04; 95% CI 1.38–3.02; *P* < .001). No other anatomical feature, including those identified as significant in the primary end point, showed a significant association with recurrence. No significant difference in recurrence was observed between the Medtronic and Boston Scientific CB systems (OR 0.90; 95% CI 0.59–1.38; *P* = .63). The absence of an orthogonal orientation of the LSPV was significantly associated with an increased risk of recurrence in both the AFA group (OR 2.74; 95% CI 1.49–5.02; *P* = .001) and the POLARx group (OR 1.83; 95% CI 1.08–3.08; *P* = .024).

#### Intra-observer variability

The mean absolute and relative difference between all repeated continuous measurements was 1.23 ± 1.28 mm. The average correlation coefficient across all variables was 0.82, with individual correlations ranging from 0.66 to 0.96. For the significant predictors of first-pass PVI success and long-term recurrence, the average correlation coefficient was 1.0 for the orthogonal orientation of the LSPV, 0.8 for the orthogonal orientation of the LIPV, and 1.0 for the presence of an RMPV.

## Discussion

This retrospective study aimed to investigate the association between LA anatomical features and both first-pass PVI success and long-term recurrence in patients undergoing CB PVI with 2 different ablation systems. We report the following findings: (1) Among all patient baseline parameters, female sex was identified as the sole predictor of first-pass PVI failure for the RSPV. (2) Among all 7 PV-specific anatomical parameters, the absence of an orthogonal orientation of the LSPV and LIPV and the presence of an RMPV for the RSPV were identified as predictors of first-pass isolation. (3) In a per-patient analysis, overall first-pass PVI with a single application was not significantly different between the 2 systems. (4) In a per-vein analysis, however, first-pass success was higher for the LIPV in the POLARx group (*P* = .03) and for the RSPV in the AFA group (*P* = .01). (5) Finally, the absence of an orthogonal orientation of the LSPV is associated with a higher risk of recurrence at 1-year follow-up.

“Single-shot” devices, such as the CB, were designed to enable PVI with a single application per vein. However, previous studies have identified an impact of anatomical features on both the short- and long-term success of the procedure.^6^, ^7^, ^8^, ^9^ To investigate the impact of the LA anatomy on both available CB platforms, we carefully selected anatomical features on the basis of previous literature demonstrating their potential association with CB PVI outcomes (Table 5). The presence of an RMPV and LCO has been linked to increased procedural complexity and a higher potential for reconnection.^6^^,^^7^^,^^12^ Further studies have identified that larger PV diameters and increased ovality reduce procedural success.^8^^,^^9^^,^^13^ Various features related to the carina, as well as the sharpness of the ridge between the LA appendage and the left-sided veins, have been linked to PVI success, with shorter and sharper features decreasing the likelihood of successful ablation.^7^^,^^14^ Additionally, the impact of PV angle and early branching on ablation effectiveness has been investigated in previous studies.^7^^,^^15^ The absence of an orthogonal orientation, often because of sharp PV angles, likely hinders optimal CB positioning. Since ice cap formation primarily occurs at the distal hemisphere of the catheter,^5^ proper isocentric balloon positioning is crucial. This central alignment enables optimal energy transfer from tissue to CB, maximizing the likelihood of successful PVI (Supplemental Figure 1).

**Table 5:** Anatomical features

Anatomical feature	AFA group (n = 96)	POLARx group (n = 95)	*P*
Additional vein	7 (7)	7 (7)	na
Left common ostium	19 (20)	19 (20)	na
Inter-carina distance			
Left (mm)	1.0 ± 1.7	1.4 ± 1.9	.16
Right (mm)	3.3 ± 2.5	4.0 ± 2.8	.09
Sharp lateral ridge	(n = 62)	(n = 62)	na
LSPV	14 (23)	11 (18)	na
LIPV	12 (19)	10 (16)	na
Ovality			
LSPV (n = 191)	75 (65–83)	73 (65–81)	.61
LIPV (n = 152)	83 (76–91)	82 (70–88)	.28
RSPV (n = 191)	86 (79–91)	84 (75–90)	.21
RIPV (n = 190)	90 (83–95)	88 (81–94)	.21
No orthogonal orientation			
LSPV (n = 191)	3 (3)	5 (5)	na
First-pass isolation rate	2 (67)	1 (20)	
LIPV (n = 152)	11 (14)	7 (9)	na
First-pass isolation rate	4 (36)	5 (71)	
RSPV (n = 191)	0 (0)	0 (0)	na
First-pass isolation rate	na	na	
RIPV (n = 190)	0 (0)	2 (2)	na
First-pass isolation rate	na	2 (100)	

Values are presented as mean ± standard deviation, median (interquartile range), or n (%).

AFA = Arctic Front Advance; LIPV = left inferior pulmonary vein; LSPV = left superior pulmonary vein; na = not applicable; Ovality = maximum diameter divided by minimum diameter; RIPV = right inferior pulmonary vein; RSPV = right superior pulmonary vein.

Although our study identified minor differences in first-pass isolation rates between the 2 cryoablation systems for the LIPV and RSPV, these effects appear to offset each other, resulting in minimal overall differences in individual efficacy. The lower first-pass PVI rates for the POLARx balloon in the RSPV might explain the increased incidence of PNP reported in earlier studies,^4^^,^^16^ as more applications with a repositioned catheter might be needed for successful PVI. Furthermore, the POLARx balloon’s compliance and flexible tip might facilitate deeper seating, especially within relatively large RSPVs, inadvertently positioning the application closer to the phrenic nerve and increasing PNP risk.^17^, ^18^, ^19^ Notably, women demonstrated significantly lower first-pass isolation rates in the RSPV, which might be attributed to the generally smaller size of the female heart^20^ and PVs, as confirmed by our data. Interestingly, established predictors such as PV ovality did not significantly affect outcomes in our cohort.^8^^,^^9^^,^^13^ This might be explained by the fact that the anatomical feature of PV orientation might have a stronger predictive power than does ovality. Furthermore, the ovality of the ostia might be better addressed by our catheter manipulation workflow than in previous studies, including those using hockey stick, push-and-pull, or rotational positioning techniques.

A nonorthogonal orientation of the LSPV and LIPV was associated with lower first-pass isolation rates and a higher risk of AF recurrence. This suggests that even with modern tools such as steerable sheaths, severe angulation of the PV ostium can hinder circumferential contact with the CB’s effective cooling zone.^5^ Given the fact that the balloon’s distal hemisphere provides cooling, an anterior orientation might prevent effective cooling, especially at the posterior wall (Graphical Abstract). This is consistent with previous literature showing that the ice cap formation with the POLARx catheter is generally smaller and less consistent than that with the AFA catheter.^5^ Although the catheter ablation of arrhythmias with a high-density mapping system in real-world practice registry reported no significant difference in long-term outcomes between patients with and without PV anatomical variants, their analysis focused only on broader variant types such as common ostia and adjacent PVs.^21^ In contrast, our findings highlight that more subtle geometric features, such as the orientation of individual veins, may still affect acute success and long-term recurrence.

Our findings underscore the potential benefits of evolving balloon technologies. Looking ahead, further innovations could include pressure-controlled cryoablation balloons designed to optimize tissue contact and energy delivery by providing real-time feedback on wall apposition. Size-adjustable CBs, such as POLARx FIT, may help address some limitations related to fixed-size balloons in variable anatomies.^22^ By enabling more proximal antrum engagement, this catheter’s design might also help reduce the risk of PNP.^23^ Additionally, incorporating temperature sensors on the balloon surface might allow direct measurement of the cooling effect and consequently increase first-pass isolation rates.^24^ Whether the reported improvements in achieving complete vein occlusion improve first-pass isolation requires further investigation.

With the emergence of pulsed field ablation (PFA) for PVI, the relevance of CB technology is being questioned.^25^ Although PFA offers a nonthermal ablation modality, some PFA systems also use balloon-based catheters, such as the novel balloon-in-basket PFA catheter highlighted in the VOLT-AF investigational device exemption trial.^26^ Thus, understanding how anatomical features, such as PV orientation and ostial dimensions, affect balloon orientation and contact, and therefore the effective heat transfer, will remain pertinent for optimizing outcomes across different balloon-based ablation platforms. Furthermore, CB PVI remains a well-established, efficient technique requiring less catheter manipulation compared with PFA, making it a straightforward procedure. Despite reports of shorter procedure times of PFA PVI compared to CB PVI,^27^ PFA is also associated with higher procedural costs, mainly because of higher equipment costs.^28^ In addition, deeper sedation or even general anesthesia is recommended, and often necessary, for PFA, especially during monopolar ablation.

In this context, optimizing CB PVI outcomes remains clinically relevant. The recognized need for further advancements in cryoablation systems underscores their potential to significantly improve PVI success and benefit patients in general.^29^ Identifying anatomical predictors of first-pass isolation can enhance procedural efficiency by reducing the number of applications and minimizing fluoroscopy and procedure times. This will also help improve safety and identify potential issues with heat transfer because of nonorthogonal vein orientations. Patient-specific tailoring of PVI procedures will be even more useful when these procedures move into more ambulatory settings.^30^

### Limitations

Analyzed imaging was either MRI or CT. With MRI being of lower image quality, it is difficult to compare both types of segmented structures. Furthermore, the lack of a standardized measurement tool gives rise to measurement errors. However, despite these potential measurement errors, the intra-observer reliability analysis indicates that the measurements were performed with sufficient consistency, thereby reducing the risk of significant measurement variability.

Even though 191 patients were analyzed, this is a relatively small study given the low prevalence of abnormal anatomical features. Finally, the small number of patients without an orthogonal vein orientation limits the statistical power for subgroup comparisons between the systems. Despite the low prevalence of nonorthogonal orientations, our findings highlight the potential impact of vein anatomy on acute and long-term CBA outcomes, underscoring the need for further studies in larger cohorts to validate these associations.

## Conclusion

Achieving optimal outcomes in PVI requires a thorough understanding of factors affecting procedural success. This study aimed to find easily extractable anatomical features that could predict procedural success. Although most anatomical features showed no strong association with first-pass isolation rates, the orientation of the left-sided PVs and the presence of an RMPV emerged as key determinants. These findings emphasize the value of preprocedural anatomical assessment using imaging modalities such as CT or MRI. Such assessment can help anticipate procedural challenges related to PV anatomy, thereby allowing physicians to tailor their procedural strategy. This might involve selecting different CB systems, if available, or using alternative modalities.
