# Supplementary material for: Impact of left atrial anatomy on pulmonary vein isolation with cryoballoon ablation: Insights from the randomized controlled COMPARE CRYO study
Source: Heart Rhythm O2. 2025 Jul 16;6(10):1499–507. doi: 10.1016/j.hroo.2025.07.005 (PMC12570213; doi:10.1016/j.hroo.2025.07.005)
Supplement: Supplementary Data [file mmc1.docx]

**Supplements**


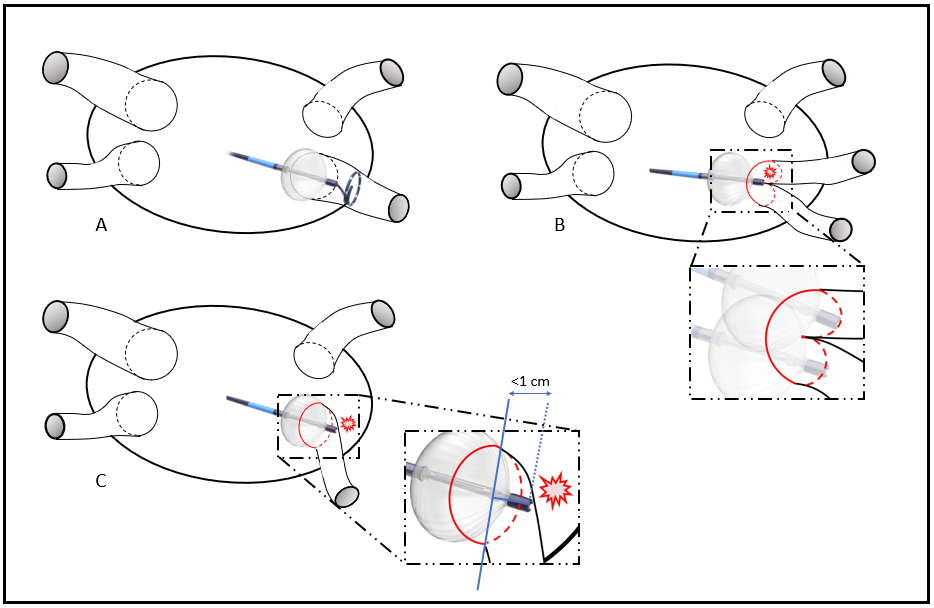
Supplemental Figure 1: Schematic visualization of exemplary situations where an orthogonal-isocentric line is absent.

A = normal anatomy; B = orthogonal orientation not present due to early branching; C = orthogonal-isocentric line not present due to sharp angle of the ostium.

Supplemental Table 1: Sub-analysis containing a logistic regression model per modality for all anatomical features on first-pass success

| ERISTICS PER VEIN | | | | | | |
| --- | --- | --- | --- | --- | --- | --- |
| Anatomical features per vein | AFA-group (n = 96) | | | PolarX-group (n = 95) | | |
|  | OR | CI (95%) | Sig | OR | CI (95%) | Sig |
| LSPV  LCO  LCD  LLRS  Ovality  Max diameter  Min diameter  No orthogonal orientation | 0.589  1.140  2.727  0.997  1.007  0.987  0.902 | 0.177 – 1.963  0.837 – 1.551  0.542 – 13.73  0.962 – 1.033  0.901 – 1.125  0.858 – 1.136  0.078 – 10.46 | 0.389  0.406  0.224  0.865  0.908  0.858  0.934 | 1.602  1.190  0.427  1.026  0.984  1.120  0.080 | 0.529 – 4.849  0.847 – 1.671  0.104 – 1.748  0.987 – 1.065  0.901 – 1.075  0.948 – 1.323  0.008 – 0.833 | 0.404  0.315  0.237  0.196  0.727  0.184  **0.035** |
| LIPV  LCO  LCD  LLRS  Ovality  Max diameter  Min diameter  No orthogonal orientation | 0.966  1.038  1.333  1.026  1.028  1.100  0.286 | 0.341 – 2.731  0.802 – 1.345  0.354 – 5.026  0.982 – 1.072  0.866 – 1.221  0.934 – 1.296  0.076 – 1.081 | 0.947  0.775  0.671  0.259  0.748  0.251  0.065 | 1.731  0.951  0.860  1.001  1.007  1.002  0.636 | 0.568 – 5.270  0.718 – 1.261  0.195 – 3.795  0.958 – 1.045  0.854 – 1.187  0.856 – 1.173  0.112 – 3.631 | 0.334  0.728  0.842  0.453  0.933  0.978  0.611 |
| RSPV  RMPV  RCD  Ovality  Max diameter  Min diameter  No orthogonal orientation (n = 0) | 3.176  1.161  1.016  0.998  1.033  na | 0.649 – 15.54  0.902 – 1.493  0.973 – 1.062  0.861 – 1.155  0.893 – 1.196 | 0.154  0.247  0.465  0.976  0.659 | 4.597  1.059  1.018  1.105  1.115  na | 0.842 – 25.09  0.906 – 1.237  0.978 – 1.058  0.970 – 1.259  0.984 – 1.264 | 0.078  0.473  0.384  0.135  0.088 |
| RIPV  RMPV  RCD  Ovality  Max diameter  Min diameter  No orthogonal orientation (n = 1) | 4.677  1.100  1.000  0.955  0.939  na | 0.857 – 25.52  0.908 – 1.332  0.952 – 1.051  0.853 – 1.068  0.830 – 1.062 | 0.075  0.330  0.994  0.418  0.315 | 1.890  0.947  0.964  1.004  0.939  na | 0.394 – 9.057  0.807 – 1.112  0.913 – 1.019  0.881 – 1.146  0.814 – 1.084 | 0.426  0.507  0.197  0.948  0.393 |
|  |  |  |  |  |  |  |

LSPV = left superior pulmonary vein; LIPV = left inferior pulmonary vein; RSPV right superior pulmonary vein; RIPV = right inferior pulmonary vein; LCO = left common ostium; LCD = left carina distance; LLRS = left lateral ridge sharpness; OIL = orthogonal-isocentric line; RMPV = right middle pulmonary vein; RCD = right carina distance.
